# The risk of serious bacterial infections among young ex-premature infants with fever

**DOI:** 10.3389/fped.2022.1021007

**Published:** 2022-10-12

**Authors:** Yuval Barak-Corren, Yoav Elizur, Shira Yuval, Amalia Burstyn, Noy Deri, Shepard Schwartz, Orli Megged, Ori Toker

**Affiliations:** ^1^ The Pediatrics Department, Shaare Zedek Medical Center, Jerusalem, Israel; ^2^ Predictive Medicine Group, Boston Children's Hospital, Boston, United States; ^3^ Faculty of Medicine, Hebrew University of Jerusalem, Jerusalem, Israel; ^4^ The Pediatric Emergency Department, Shaare Zedek Medical Center, Jerusalem, Israel; ^5^ The Pediatric Infectious Disease Unit, Shaare Zedek Medical Center, Jerusalem, Israel; ^6^ The Allergy and Clinical Immunology Unit, Shaare Zedek Medical Center, Jerusalem, Israel

**Keywords:** invasive bacterial infection (IBI), severe bacterial infection (SBI), neonatal fever, sepsis workup, premature infants

## Abstract

**Background and Objectives:**

To determine the rate of serious-bacterial-infections (SBI) in young ex-premature infants with fever, and to develop a risk-stratification algorithm for these patients.

**Methods:**

A retrospective cohort study including all infants who presented to the pediatric emergency department (ED) of a tertiary-care university-hospital between 2010 and 2020 with fever (≥38°C), were born prematurely (<37-weeks), had post-conception age of <52-weeks, and had available blood, urine, or CSF cultures. The rates of SBI by age-of-birth and age-at-visit were calculated and compared to a cohort of matched full-term controls.

**Results:**

The study included a total of 290 ex-premature cases and 290 full-term controls. There were 11 cases (3.8%) with an invasive bacterial infection (IBI) of either bacteremia, meningitis or both and only six controls (2.1%) with IBI (*p* = 0.32). Over 28-days chronologic-age, there were 10 (3.6%) IBIs among cases and no IBIs among the controls (*p* = 0.02). There were eight (3%) cases and three (1%) controls with IBI who were well-appearing on physical examination (*p* = 0.19). All eight well-appearing ex-premature infants were under 60-days adjusted-age, seven of whom (88%) were also under 28-days adjusted-age. There were 28 (10.6%) cases and 34 (12%) controls with urinary tract infection (UTI) (*p* = 0.5). Among cases under 60-days adjusted-age, urinalysis was not reliable to exclude UTI (50% negative).

**Conclusions:**

Well-appearing ex-preterm infants have a significant risk for IBI until the adjusted age of 28-days and for UTI until the adjusted age of 60-days. Further studies are needed to evaluate the approach to fever in this unique population.

## Introduction

Worldwide, approximately 15 million infants are born prematurely every year (1). The rise in incidence of premature births in recent years coupled with improved postnatal care, accounts for premature infants representing a growing percentage of the pediatric population (2). As with term-infants, these premature-infants may develop fever in the weeks following delivery. Fever in a young infant, whether born at term or prematurely, poses a significant diagnostic dilemma since the ability to detect all serious bacterial infections (SBI) based on the history, physical examination and laboratory exam findings in this age group remains challenging (3). Because missed SBIs, particularly bacteremia and meningitis, may lead to serious complications, the management of young febrile infants frequently requires lumbar puncture, empiric broad-spectrum antibiotic administration, and hospitalization.

With the goal of identifying which febrile infants are at higher risk for SBI and thus warrant these interventions, as well as those at lower risk who could be safely managed without them, widely accepted clinical guidelines applicable to specified age groups during the first three months of life have been developed (4, 5). These guidelines exclude infants with a background of prematurity who are often automatically classified as being at high risk for SBI, perhaps unnecessarily, and regardless of their specific medical history or week of birth (6–8). Recommendations for the management of premature infants with fever in the immediate post-partum period are well established (9). Lacking though, are evidence-based guidelines for the management of infants who develop fever after they have already been discharged from the Neonatal Intensive Care Unit (NICU). The only previous study to address this issue found that the rate of SBI among ex-premature infants was not higher than that of full-term infants (10).

The aim of our study was to evaluate the risk for SBI among ex-premature infants with fever and a post-conception age of less than 52 weeks and to compare this risk to that of full-term infants. We also sought to evaluate the implication of applying the new American Academy of Pediatrics (AAP) guidelines for the “Evaluation and Management of Well-Appearing Febrile Infants 8 to 60 Days Old” (11) to this unique cohort of patients which was excluded from these guidelines. Finally, we aimed to explore if it is necessary to account for the adjusted age (the age the infant would be if born at 40 weeks of gestation) of the ex-premature febrile infant when applying these clinical criteria, and if so, until what chronologic and adjusted ages.

## Methods

A single center retrospective cohort analysis was conducted at a 1,000-bed tertiary care teaching hospital with a pediatric ED visit rates of approximately 35,000 children per year, and with more than 22,000 newborn deliveries per year. The electronic medical records of all ED visits between April-2010 and April-2020 of infants with a history of admission to the NICU, who presented to the ED during the first year of life, and for whom there was any documentation of fever and/or availability of body fluid culture were reviewed. Inclusion criteria were: 1. History of prematurity: gestational age (GA) of less than 37-weeks; 2. Fever: body temperature of 38°C or greater, measured either at home or in the ED; and 3. Age: adjusted age of less than three months (i.e., less than 52-weeks from conception). Exclusion criteria were: 1. Lack of blood, urine, and cerebrospinal fluid (CSF) cultures; 2. Existence of a foreign medical device such as a ventriculoperitoneal (VP) shunt or central line; and 3. A known immunodeficiency.

Based on this cohort of ex-premature infants with fever, a control-cohort of full-term infants with fever was extracted. Patients were eligible as controls if they arrived at the ED with fever (measured >38°C), were known to be born at term (documented GA ≥ 37-weeks), had a chronologic age of less than 12-weeks and arrived at the ED on the same days as the case-subjects (< 1 day difference). The same exclusion criteria as above were applied. From this cohort of potential controls, we randomly selected a representative sample of equal size to that of the cases-cohort.

Data on demographics, vital signs, physical appearance, laboratory tests, co-morbidities and chronic diseases, and major complications associated with the NICU hospitalization (for cases) were collected for each included patient (Supplementary Appendix 1). A urine dipstick was considered positive if leukocyte-esterase and/or nitrites were identified. Urine cultures were obtained either *via* suprapubic aspiration (SPA), in-out catheter, or clean-catch. Cultures were considered positive if at least 100 colony forming units (CFU) per ml were grown from SPA, >10,000 CFU/ml from catheter, or >100,000 CFU/ml from a clean catch, and only if there was growth of a single pathogen (or two pathogens both typical for UTI).

The main outcome measure was the rate of SBI among the study cohort. Separate analyses were conducted to assess the rate of invasive bacterial infections (IBI, i.e., bacteremia and/or meningitis) and UTI. The rates of SBI and IBI were measured separately for both cases and controls, and the two cohorts were compared to search for statistically significant differences in their rate (SBI = IBI ± UTI). We compared the rates of SBI and IBI both for the entire cohorts as well as for each age group separately (by age in months). Finally, we retrospectively applied the new American Academy of Pediatrics (AAP) guidelines for the “Evaluation and Management of Well-Appearing Febrile Infants 8 to 60 Days Old” to both cohorts to test the implications of using these guidelines on this population of infants with a background of prematurity (11). For all age-dependent analyses, the cases of prematurity were assessed both by their chronologic age as well as their adjusted age (the age the infant would be if born at 40 weeks of gestation).

All continuous variables were measured for normality using the Shapiro-Wilk Normality Test. Normally continuous variables were presented as mean with 95% CI and non-normally distributed variables with median and interquartile range (IQR). The comparison of continuous variables was performed using the student t-test or Wilcoxon Rank Sum depending on whether the variable was normally distributed, or not. Categorical variables were compared using a chi-square test. All analyses were conducted using R statistical software (12). The study was approved by the medical center's Review Board.

## Results

### Cohort of cases: patients with a history of prematurity

**Figure 1:**
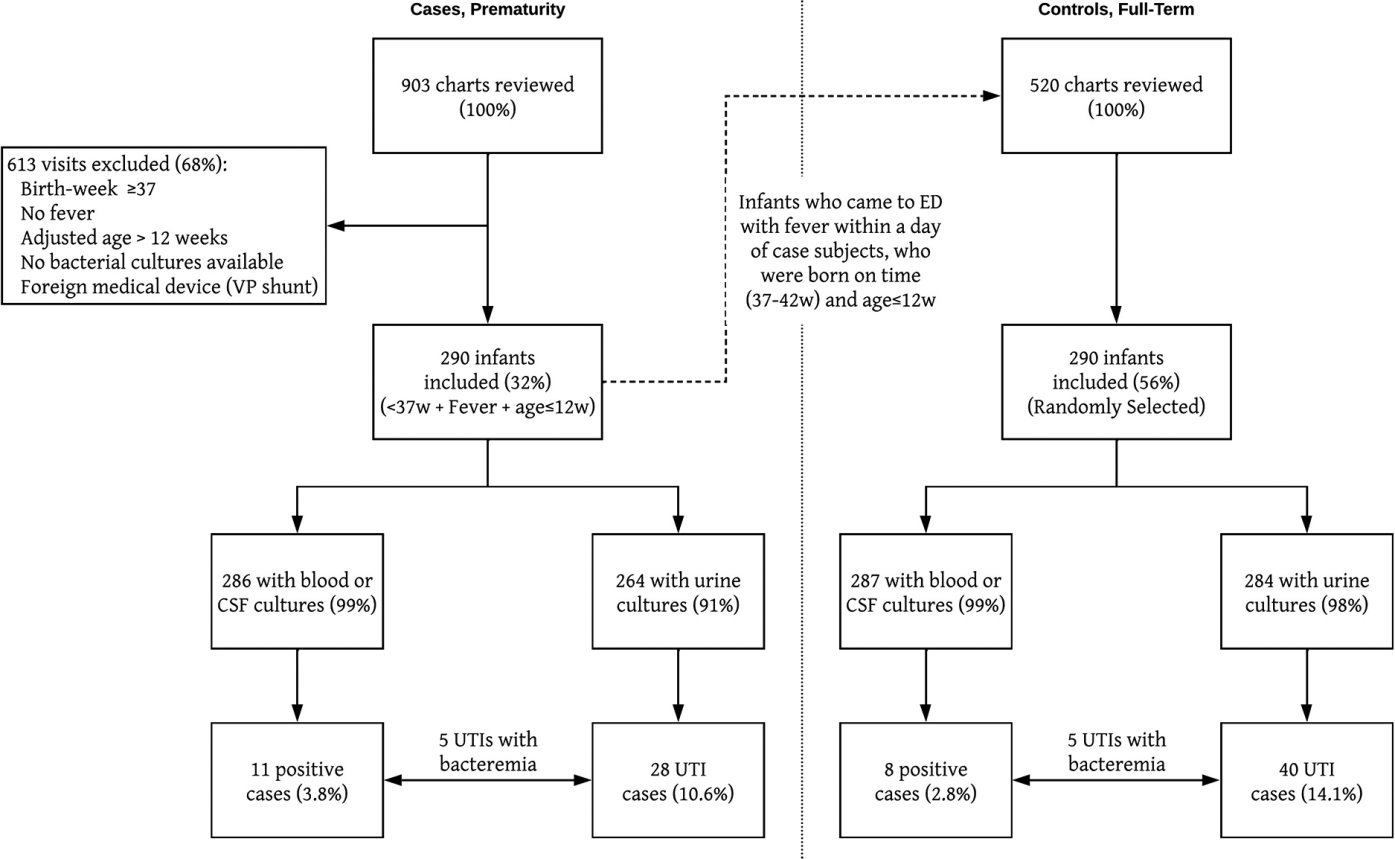
Flow-chart diagram of study design, data availability and culture results.

A total of 903 medical records were reviewed and 297 (33%) infants met the inclusion criteria. Seven infants were excluded due to unavailable culture results (*n* = 5) or the presence of a foreign medical device (VP shunt, *n* = 2). Of the two hundred and ninety (32%) infants included in the study, 56% (*n* = 163) were male (Figure 1). The median GA at birth was 34 weeks, with a range of 24 to 36 weeks and with the following distribution: 10.7% born at GA of 24–28 weeks (*n* = 31), 25.9% born at GA 29–32 (*n* = 75), and 63.4% born at GA 33–36 (*n* = 184). The median chronologic and adjusted ages at the time of the ED visit was 10.6 weeks (IQR 7.3–15.3 weeks) and 3.5 weeks (IQR 1.1–7.1 weeks), respectively (Table 1).

**Table 1:** Summary statistics for the 290 cases and 290 controls included in the study.

Feature	Cases (*n* = 290)	Controls (*n* = 290)	*p*-value
Sex			1
Female	43.8% (*n* = 127)	43.8% (*n* = 127)	
Male	56.2% (*n* = 163)	56.2% (*n* = 163)	
Birth week (median and IQR)	34 [31–35]	39 [39–40]	<0.001
Weeks 24-28	10.7% (*n* = 31)	0% (*n* = 0)	
Weeks 29-32	25.9% (*n* = 75)	0% (*n* = 0)	
Weeks 33-36	63.4% (*n* = 184)	0% (*n* = 0)	
Chronologic age (weeks)*	10.6 [7.3–15.3]	5 [3–7]	<0.001
Adjusted age (weeks)*	3.5 [1.1–7.1]	5 [3–7]	<0.001
**Blood cultures**			
Total cultures	98.6% (*n* = 286)	99% (*n* = 287)	1
Positive cultures	3.5% (*n* = 10)	2.1% (*n* = 6)	0.443
**Urine cultures**			
Total cultures	91% (*n* = 264)	97.9% (*n* = 284)	<0.001
Positive cultures	10.6% (*n* = 28)	12% (*n* = 34)	0.712
**CSF cultures**			
Total cultures	37.6% (*n* = 109)	52.8% (*n* = 153)	<0.001
Positive cultures	2.8% (*n* = 3)	1.3% (*n* = 2)	0.701
Blood, urine, and CSF cultures available	37.2% (*n* = 108)	51.7% (*n* = 150)	0.001
Serious Bacterial Infections (SBI)	11.7% (*n* = 34)	12.8% (*n* = 37)	0.8
Invasive Bacterial Infections (IBI)	3.8% (*n* = 11)	2.1% (*n* = 6)	0.325

* Median age at ED presentation [IQR].

### Cohort of controls: patients born at term

**Figure 2:**
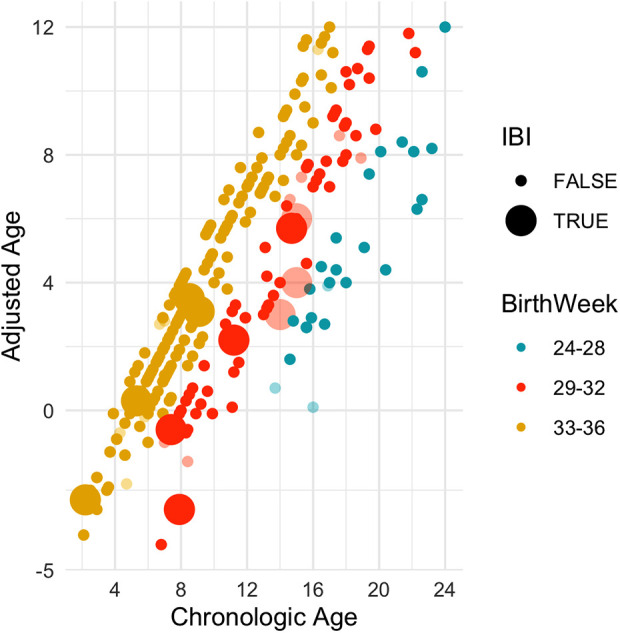
A scatter plot of all 290 included cases. Faded out are infants who were ill appearing to begin with. The observations are shown by the adjusted-age (y-axis) and chronologic-age (x-axis) in weeks. Enlarged icons indicate a positive culture. As can be seen, the IBI cases continue to be prevalent beyond 60 days chronologic-age, but almost all cases fall under 28 days adjusted age.

A total of 520 medical records were found eligible for inclusion as controls from which we randomly selected a sample of 290 records to match the cases (Figure 1). The male/female ratio was equal to that of the cases (56% and 44% respectively). The median GA at birth was 39 weeks (range 37–42). The median age at the time of the ED visit was 5 weeks (IQR 3–7) was significantly lower than the 10.6 weeks of the cases (*p* < 0.001, Table 1), and there were significantly more controls under 28 days of age at the time of the ED visit than cases (*n* = 116 vs. *n* = 12, *p* < 0.001, Figure 2).

### All serious bacterial infections (SBI)

Of the 290 premature-cases, 34 (11.7%) had a positive bacterial culture of either blood, urine, or CSF. There were 28 infants who were ill-appearing according to the treating physician in the ED, of whom five (18%) had an SBI. The SBI rate among the well-appearing infants was thus 11% (29/262). A scatter plot of all SBI cases in the cases-cohort, by chronologic and adjusted age, is shown in Supplementary Figure S1. Of the 290 controls, 37 (12.8%) had a positive bacterial culture of either blood, urine, or CSF. There were 16 infants who were ill-appearing according to the treating physician in the ED, of whom five (31.2%) had an SBI. The SBI rate among the well-appearing infants was 11.7% (32/274) with 90.6% of these (29/32) being a UTI only. There was no statistically significant difference in the overall SBI rates between cases and controls (*p* = 0.8).

### Bacteremia and meningitis (IBI)

Of the 290 case-subjects included, blood or CSF culture results were available for 286 (98.6%). Bacteremia was detected in 10 infants, three of whom also had bacterial meningitis. One infant had bacterial meningitis without bacteremia. Thus, a total of 11 infants (3.8%) had an invasive bacterial infection (IBI) of either bacteremia, meningitis, or both (Table 1). A comparison of various relevant clinical parameters of these 11 infants with those of ex-premature infants whose blood, CSF and urine cultures were negative revealed that the percentage of neutrophils in the CBC was higher in the former group (50% vs. 40%, *p* = 0.01). The CRP was also higher in the IBI group, yet only 5/11 IBI cases had available CRP results and this difference was not statistically significant (4.5 vs. 1.1 mg/dl, *p* = 0.17). There were no other statistically significant differences between the two groups (Supplementary Appendix 3, Supplementary Table S1).

The rate of bacteremia and meningitis varied by both GA at birth and age at presentation to the ED (Figure 2). The rate was highest among the middle group of 29–32 weeks GA (9%, *n* = 7/74), with 0% among infants born <29 weeks GA and only 2% among infants born >32 weeks (OR = 5.4, 95% CI 1.5–19.1, *p* = 0.01). There were eight infants with IBI who were well appearing on initial impression. Of these, three were over the chronologic age of 60 days and thus would not be addressed by the standard AAP guidelines for the evaluation of well-appearing infants with fever (Figure 2 and Supplementary Appendix 3 Supplementary Figure S3). All eight infants were under the adjusted age of 60 days, seven of whom (88%) were under the adjusted age of 28 days. The one infant who was 29–60 days old (40 days old) had an abnormal urinalysis and was found to have E. Coli UTI and bacteremia (Supplementary Appendix 3 Supplementary Table S2 and Supplementary Figure S4).

**Figure 3:**
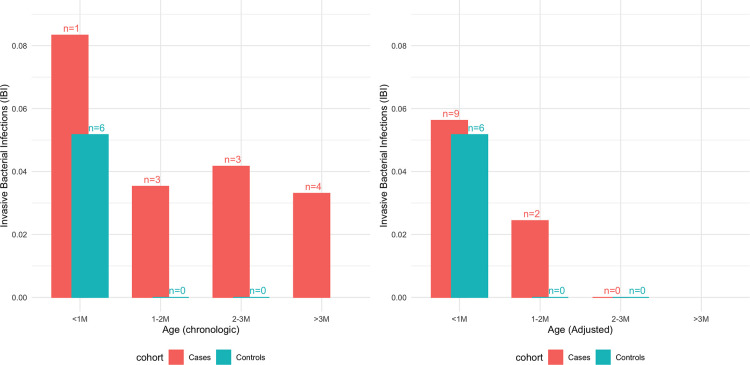
The rate of invasive bacterial infections (IBI) among ex-premature cases and full-term controls by age in month, using only chronologic ages (left) and chronologic age for controls and adjusted age for cases (right).

The term-infants had a lower rate of IBI than the cases (Figure 3), yet this was not statistically significant (2.1% vs. 3.8%, *p* = 0.32). The difference was more pronounced when including only the well-appearing infants (1% vs. 3%, *p* = 0.19) and it became significant on analysis of infants who were older than 28 days old (chronologic age), well appearing or not, with 0% IBIs among controls vs. 3.6% (*n* = 10) IBIs among cases (*p* = 0.02) (Supplementary Appendix 3, SupplementaryFigures S3 and S5). There was no statistically significant difference in the IBI rate once adjusting the age of the ex-premature infants (0% vs. 1.5%, *p* = 0.36). Like the ex-premature infants, both CRP and neutrophils count were elevated among controls with IBI compared to controls with sterile cultures: median CRP of 3.4 mg/dl (IQR 0.6–6.6) vs. 0.6 mg/dl (IQR 0.2–1.9, *p* = 0.05) and median neutrophil count of 8,000 (IQR 3,200–11,300) vs. 3,500 (IQR 2,300–5,200, *p* = 0.18) (Supplementary Appendix 3 Supplementary Table S1).

### Urinary tract infections

Urine culture results were available for 264 (91%) of the 290 case-infants, of which 28 (10.6%) were positive. There were 23 cases of an isolated UTI and five cases of UTI with bacteremia (included in the previous IBI analysis). Sixty-four percent (*n* = 18) of the positive cultures were obtained *via* in-out catheter, 32% (*n* = 9) *via* suprapubic aspiration and one culture *via* clean-catch. In contrast to the 11 cases of IBI, the prevalence of the isolated UTI cases was statistically similar between the three GA groups with a rate of 17%, 9%, and 10% for infants born at GA 24–28, 29–32, and 33–36 weeks, respectively (*p* = 0.5). The UTI rate also did not vary with the chronologic age of the infant (Supplementary Appendix 3, Supplementary Figure S2). A detailed summary of all UTI cases appears in Supplementary Appendix 3, Supplementary Table S3. As expected, UTIs were more common among females than males by a ratio of 2.5:1, yielding an odds ratio of 3.3 (95% CI 1.4–7.9). In the 25 cases for which urine dipstick results were available, 11 (44%) did not show signs of infection (no leukocyte esterase or nitrites). Based only on the dipstick, eight UTI cases would have been missed all of whom were under 60-days adjusted age (50% of all UTIs in this age-group, Supplementary Table S3). The controls had a slightly higher rate of UTI compared to the cases, but this was not statistically significant (12% vs. 10.6%, *p* = 0.5). Interestingly, in the control cohort UTIs were slightly more common among male patients than female patients (OR of 0.75, 95% CI 0.36–1.56), yet this was not statistically significant. The rate of normal dipstick in patients with UTI was slightly lower than that seen in the cohort of cases (*n* = 9, 26% vs. 44%, *p* = 0.26).

## Discussion

In this study, we reviewed the medical records of 290 ex-premature infants with an adjusted age of less than three months, and 290 full-term infants with a chronological age of less than three months, who presented to the ED with fever. We did not find a statistically significant difference in the rate of serious bacterial infections between infants with a background of prematurity and infants born at term, yet the ex-premature infants were older than the controls with a median chronologic age of 10.6 weeks vs. 5 weeks for controls. When only considering cases and controls over the age of 28-days (chronologic age), we found that the cases had a significantly higher rate of IBIs compared to the controls (3.6% vs. 0%, *p* = 0.02). Overall, we found that infants who were born prematurely are at an increased risk for IBI until the adjusted age of 60 days, highlighting the need to manage these patients with caution. Further discussion on the significance of some of the ancillary laboratory tests can be found in the supplementary material (Supplementary Appendix 4).

To date, research on the evaluation of fever in ex-premature infants has primarily focused on the SBI rate during the NICU hospitalization (13, 14). Inoue et al. examined the prevalence of SBI among 141 ex-preterm infants with a post-conception age of less than 48 weeks who were evaluated in the ED. (9) They found that the rate of SBI in these infants was 9.2%, similar to the previously reported SBI-rate in febrile full-term infants (Supplementary Appendix 3, Supplementary Table S4). While the overall SBI rate in our cases cohort was similar to that found by Inoue et al. (11.6% vs. 9.2%, *p* = 0.55), we demonstrate that this rate varies significantly with the GA at birth and with the chronologic and adjusted ages at the time of the ED visit. These findings are especially important when considering age adjustment for these patients. Importantly though, Inoue et al. included in their analysis infants who underwent evaluation for fever or for other symptoms (ex: apnea), while we only included infants who underwent evaluation due to fever. We hope that the data gathered in our study will help to provide a more focused guide for clinicians managing fever in this unique population.

This study has several limitations. The relatively small sample size of 580 infants limits the generalizability of our findings. Yet, as the first of its kind, our study can serve as a model for others which may corroborate our results. Second, since cultures of blood, urine and CSF were not obtained from all infants, it is possible that cases of meningitis, bacteremia or UTI were not detected, leading to an underestimation of the true rate of SBI. Nonetheless, blood cultures were almost always available (99%) and our review of each infant's medical record did not reveal a subsequent ED visit leading to a diagnosis of an SBI that was missed on the index ED visit. Similarly, inflammatory markers were not universally available, thus limiting our ability to apply the recent AAP's risk stratification to these patients. Third, our study population represents infants admitted to the ED for evaluation of fever and our results may not be fully applicable to the outpatient setting. Certainly though, the significant rate of SBIs which we observed among otherwise well-appearing low-risk infants should alert the outpatient clinician to manage these infants with particular caution.

To the best of our knowledge, a study to assess the prevalence of SBI and IBI in a cohort of ex-premature infants evaluated in the ED exclusively for fever has not been previously reported. We found that febrile ex-preterm infants who are both well-appearing and otherwise at low risk for SBI still have a significant rate of IBI until the adjusted age of 28 days and of UTI until the adjusted age of 60 days. Additionally, since the urine dipstick test was found to be unreliable in its ability to detect a UTI in this age group, and since the rate of UTIs continued to be significant until the adjusted age of 60 days, we recommend that clinicians maintain a high level of suspicion for UTI in this population. Further prospective and multi-site studies are warranted to confirm these findings.

## Data Availability

The original contributions presented in the study are included in the article/**Supplementary Material**, further inquiries can be directed to the corresponding author/s.
