# Supplementary material for: The risk of serious bacterial infections among young ex-premature infants with fever
Source: Front Pediatr. 2022 Oct 12;10:1021007. doi: 10.3389/fped.2022.1021007 (PMC9597199; doi:10.3389/fped.2022.1021007)
Supplement: Supplementary file 1 [file DataSheet1.docx]

**Supplementary Material**

**Appendix 1 – Data Collection**

The following data were collected for each study patient: GA at birth (by week); age (chronologic and adjusted, by week) on the day of the ED visit; sex; vital-signs (temperature, heart rate, oxygen saturation); chief complaint; general appearance (as documented in the chart by the treating physician); pertinent physical exam findings; screening laboratory tests including complete blood count (CBC), C-reactive protein (CRP) and urine dipstick results; the microbiology culture results (of blood, urine, CSF, and other body fluids if obtained); and treatment disposition regarding administration of antibiotics and hospitalization. If, for any of the above, multiple measures were available, the first was used. In addition to the above information, details of each patients’ medical course during their stay in the NICU after birth, including prior infections, prior surgeries, and any prematurity-associated complications were collected.

**Appendix 2 – The Israeli guidelines for the management of febrile infants 0 to 3 months of age in the Emergency Department.** Assembled by the Israel Pediatric Society and the Israeli Medical Association (July 2018).

1. All infants presenting with a toxic appearance should undergo a full-sepsis workup, including collections of blood, urine and CSF cultures, followed by hospitalization with empiric IV antibiotics treatment.
2. All neonates (age <1 month) with fever should undergo a full-sepsis workup and inpatient antibiotics treatment.
3. Febrile infants 1 to 2 months of age at high risk^^[[1]](#footnote-1)^^ for SBI should undergo a full sepsis workup and inpatient antibiotics treatment.
4. Febrile infants 1 to 2 months of age at low-risk for SBI (meet none of the high-risk criteria) can be discharged without antibiotic prescription, as long as careful outpatient follow-up will be done.
5. Febrile infants 2 to 3 months of age at high-risk for SBI should be considered for full sepsis workup, including LP. Yet, based on clinical judgments such patients can be hospitalized or discharged for outpatient follow-up after receiving 3^rd^ generation-cephalosporins (e.g, Ceftriaxone).

**Appendix 3 – Additional tables and figures**

| **Table S-1:** Comparison of demographic features, vital signs, and lab tests among three sub-cohorts: infants with bacteremia/meningitis, UTI only, or all sterile-cultures. Top – for the *ex-premature cases cohort and Bottom – for the full-term controls.* | | | | | | |
| --- | --- | --- | --- | --- | --- | --- |
| **Ex-Premature Cases** | | | | | | |
|  | | **Bacteremia or Meningitis (n=11)** | | **UTI (n=23)** | | **Sterile Culture (n=256)** |
| Birth week | 31 [30-34.5], p=0.13 | | 34 [29.5-35], p=0.5 | | 34 [31.8-35] | |
| Chronologic age (week) | 9.1 [7.7-14.3], p=0.56 | | 12.8 [8.2-15.1], p=0.46 | | 10.5 [7.3-15.5] | |
| Adjusted age (week) | 3 [-0.1-3.8], p=0.09 | | 3.9 [1.2-6.6], p=0.97 | | 3.7 [1.2-7.2] | |
| Vital signs |  | |  | |  | |
| Temperature (c˚) | 38.4 [38.3-39.1], p=0.12 | | 38.3 [37.8-38.8], p=0.27 | | 38.3 [38-38.8] | |
| Heart rate | 177.1 [157.5-196.6], p=0.25 | | 161.9 [153.9-169.9], p=0.28 | | 166.3 [164.2-168.5] | |
| O2 Saturation (%) | 97.5 [97-99.8], p=0.24 | | 99 [97-100], p=0.08 | | 98 [95-99] | |
| Labs |  | |  | |  | |
| WBC (10^3^/ul) | 10.6 [4.8-13.9], p=0.41 | | 10.7 [8.7-13.4], p=0.86 | | 10.4 [7.5-14.3] | |
| % NEUT | **0.5 [0.4-0.5], p=0.01** | | 0.3 [0.3-0.4], p=0.35 | | 0.4 [0.3-0.5] | |
| ABS NEUT (10^3^/ul) | 4.7 [2.2-6.6], p=0.45 | | 3.5 [2.7-4.6], p=0.95 | | 3.4 [2.3-5.2] | |
| PLT (10^3^/ul) | 358 [264.7-451.3], p=0.18 | | 435.7 [375.9-495.5], p=0.6 | | 419.4 [400.3-438.5] | |
| CRP (mg/dL) | 4.5 [1.5-5.3], p=0.17 | | 1.4 [0.2-1.8], p=0.79 | | 1.1 [0.5-2.6] | |
| **Full-Term Controls** | | | | | | |
|  | | **Bacteremia or Meningitis (n=6)** | | **UTI (n=31)** | | **Sterile Culture (n=253)** |
| Birth week | 39.5 [39-41.5], p=0.39 | | 39 [38-40], p=0.1 | | 39 [39-40] | |
| Chronologic age (week) | **1.8 [1.7-3], p=0** | | **3.3 [2.2-7], p=0.04** | | 5 [3.3-7.1] | |
| Adjusted age (week) | **1.8 [1.7-3], p=0** | | **3.3 [2.2-7], p=0.04** | | 5 [3.3-7.1] | |
| Vital signs |  | |  | |  | |
| Temperature (c˚) | 39.1 [38.8-39.3], p=0.07 | | 38.6 [38.3-39], p=0.72 | | 38.6 [38.3-39] | |
| Heart rate | 182 [161.4-202.6], p=0.12 | | 164.9 [156.5-173.2], p=0.6 | | 167.1 [164.8-169.5] | |
| O2 Saturation (%) | 95.5 [95-97.5], p=0.22 | | 97 [96-99], p=0.95 | | 97 [95.2-99] | |
| Labs |  | |  | |  | |
| WBC (10^3^/ul) | 14.3 [6.6-16.4], p=0.43 | | **13.7 [10.8-20.2], p=0** | | 9.7 [6.9-12.6] | |
| % NEUT | **57.9 [39.1-73], p=0.05** | | **54.6 [45-65.4], p=0** | | 38.9 [28.3-50.5] | |
| ABS NEUT (10^3^/ul) | 8 [3.2-11.3], p=0.18 | | **6.7 [4.2-13.3], p=0** | | 3.5 [2.3-5.2] | |
| PLT (10^3^/ul) | 352.5 [156.9-548.1], p=0.89 | | 379.2 [331.7-426.7], p=0.54 | | 363.9 [347.9-379.8] | |
| CRP (mg/dL) | **3.4 [0.6-6.6], p=0.05** | | **4.9 [1.6-8.2], p=0** | | 0.6 [0.2-1.9] | |
| All variables except for ‘Heart Rate’ and ‘Platelets’ have a non-normal distribution thus we show the median and IQR and p-values are calculated using Shapiro-Wilk test. For heart rate and platelets we show mean and 95% CI, and p-value is calculated using student t-test. All values are compared to the control group of sterile-cultures. Chronological and adjusted ages are shown in weeks. | | | | | | |

**Table S-2**: Demographic and clinical features of all invasive bacterial infection cases (bacteremia and/or meningitis).

| **Age (weeks)** | | |  |  | **Labs** | | | | |  | **Cultures** | | | |
| --- | --- | --- | --- | --- | --- | --- | --- | --- | --- | --- | --- | --- | --- | --- |
| **BW** | **CHR** | **ADJ** | **Sex** | **Appear** | **WBC**  (10^3^/µL) | **% Neut** | **Abs Neut**  (10^3^/µL) | **CRP**  (mg/dL) | **Dipstick** | **Temp**  (°C) | **Urine** | **Blood** | **CSF** | **O** |
| 29 | 7.9 | -3.1 | M | NA | 3.6 | 58% | 2.09 | 1.5 | INF | 38.4 |  | GBS |  |  |
| 35 | 2.2 | -2.8 | M | Well | 14.6 | 46% | 6.72 |  | NA | 38.4 | E. Coli | E. Coli |  |  |
| 32 | 7.4 | -0.6 | M | Well | 3.8 | 39% | 1.5 | 0.2 | Normal | 38.4 |  | GBS | GBS |  |
| 35 | 5.3 | 0.3 | M | Well | 16.4 | 46% | 7.5 | 4.5 | INF | >38 | E. Coli | E. Coli |  |  |
| 31 | 11.2 | 2.2 | M | Well | 8.7 | 47% | 4.07 | (19.1)^¥^ | NA | 38.3 |  | S. Aure |  | ¥ |
| 29 | 14 | 3 | M | Not Well | 13.2 | 49% | 6.45 |  | Normal | 38.3 | Cont | S. Pneu. |  |  |
| 34 | 9.1 | 3.1 | F | Well | 15.5 | 58% | 8.99 |  | INF | 38.9 | K. Pneum. | K. Pneu. |  |  |
| 35 | 8.5 | 3.5 | F | Well | 10.6 | 61% | 6.47 |  | Normal | 39.8 |  | S. Pneu. |  | † |
| 29 | 15 | 4 | F | Not Well | 2.4 | 26% | 0.61 | 14.4 | INF | 39.3 | E. Coli + E. Faecalis | S. Pneu. | S. Pneu. |  |
| 31 | 14.7 | 5.7 | F | Well | 5.9 | 40% | 2.36 |  | INF | 39.5 | E. Coli | E. Coli | Not Taken |  |
| 31 | 15 | 6 | M | Not Well | 10.9 | 43% | 4.72 | 5.3 | Normal | 38.6 |  |  | N. Meningitis |  |
| Abbreviations: Age: BW = birth-week (gestational age at), CHR = chronologic age, ADJ = adjusted age; Sex: M = male, F = female; Appear: overall appearance in the PED, NA = not available; Labs: Neut = Neutrophiles, CRP = C-Reactive Protein, INF = signs of infection on Dipstick (either nitrites or leukocyte esterase); Cultures: CSF = cerebrospinal fluid, O = Other; Bacteria: E. Coli = *Escherichia coli*, GBS = *Group B Streptococcus*, S. Aureues = S*taphylococcus aureus*, S. Pneum. = *Streptococcus pneumoniae*, K. Pneum. = *Klebsiella pneumoniae*, N. Meningitis = *Neisseria meningitidis*, E. Faecalis = *Enterococcus faecalis*.  ¥ An infant with septic-arthritis. CRP was not taken in the initial evaluation at the PED, it was only taken two days later when cultures returned positive for *staphylococcus aureus.* The arthritis was only noticed one day into hospitalization. † Acute otitis media caused by *Streptococcus pneumoniae*. | | | | | | | | | | | | | | |

**Figure S-1**: Scatter plot of all included patients with either blood, urine or CSF cultures available, shown by the adjusted-age (y-axis) and chronologic-age (x-axis), and grouped by the gestational week at birth (**Yellow**: weeks 33-36, **Red**: weeks 29-32, **Blue**: weeks 24-28). Patients with a positive culture shown with enlarged icons. Patients who were ill-appearing to begin with, are shown in faded Gray. Plot (A) for any positive culture: urine, blood, or CSF (i.e. SBI); and plot (B) for UTIs only.


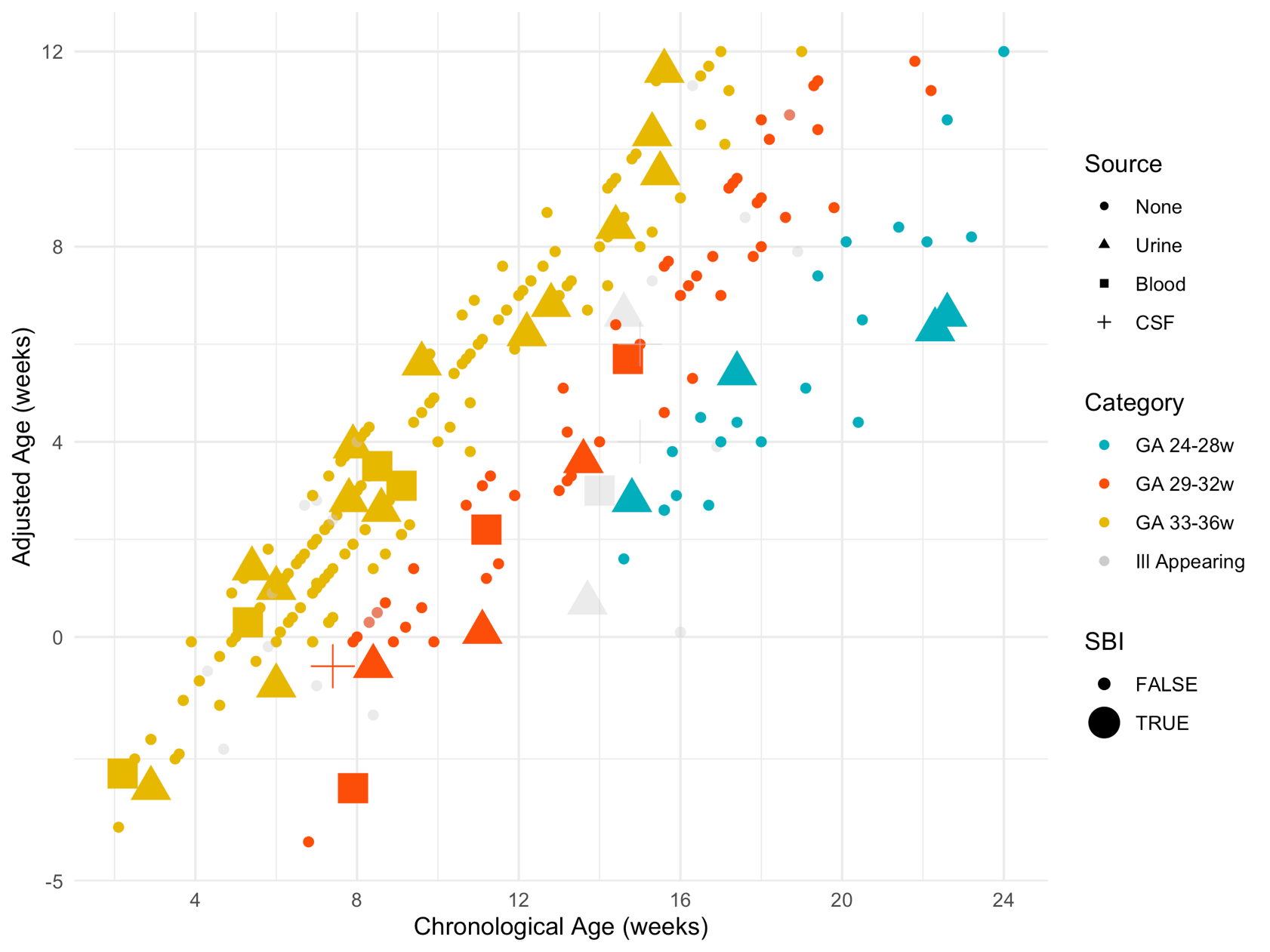


**A.**

.

**
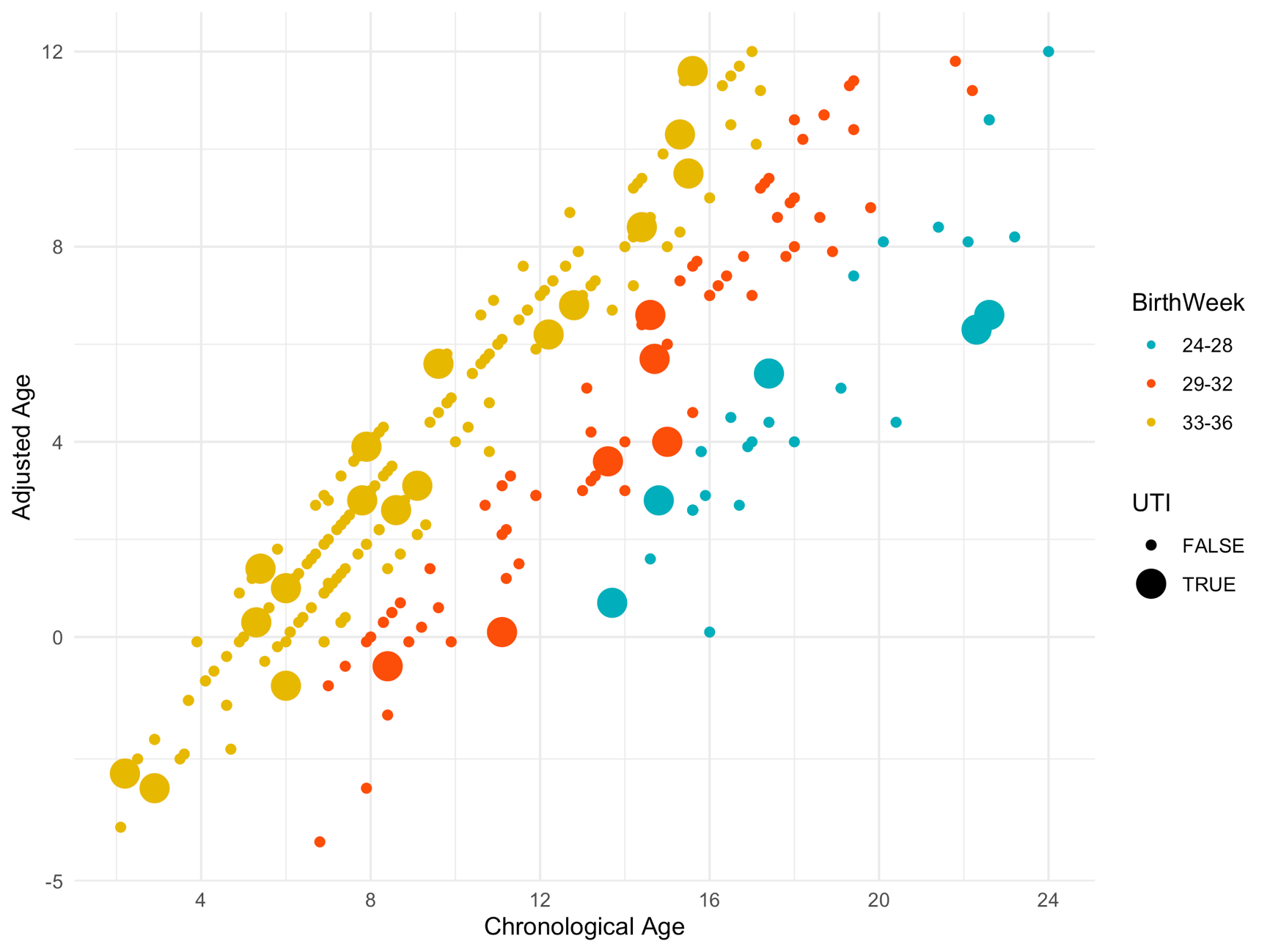
**

**B.**

Figure S-2: Retrospective application of the new (2021) AAP guidelines for the “Evaluation and Management of Well-Appearing

Febrile Infants 8 to 60 Days Old” over the cohort of ex-premature cases using their chronologic age. ^§^ Any one of the following: Fever > 38.5°C, CRP > 2.5 mg/dL, or absolute neutrophile count > 4,000 mm^3^. The figure includes 3 applications of the algorithm: (A) for ex-premature infants, by chronologic age; (B) for ex-premature infants, by adjusted age; and (C) for the full-term controls cohort.


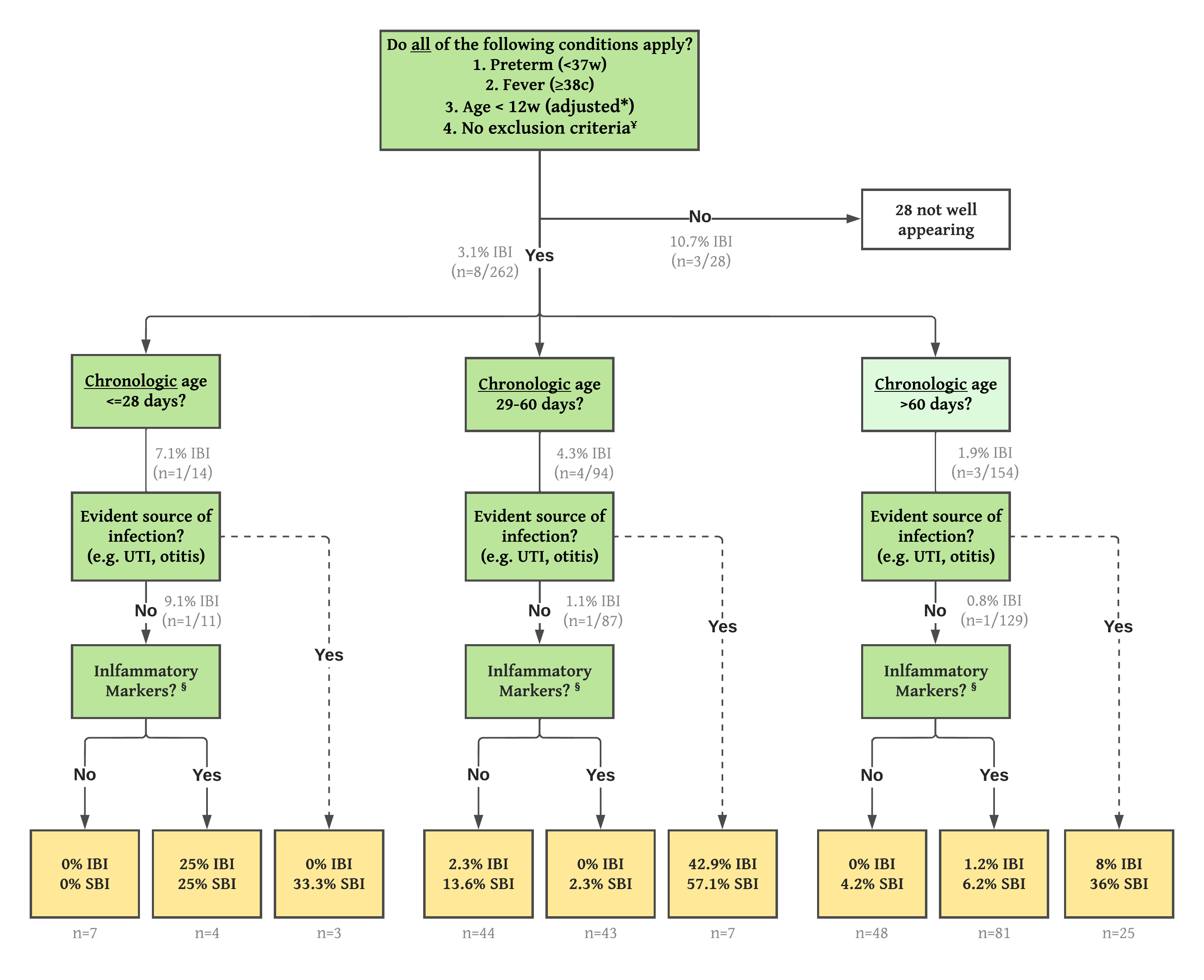


**A.**


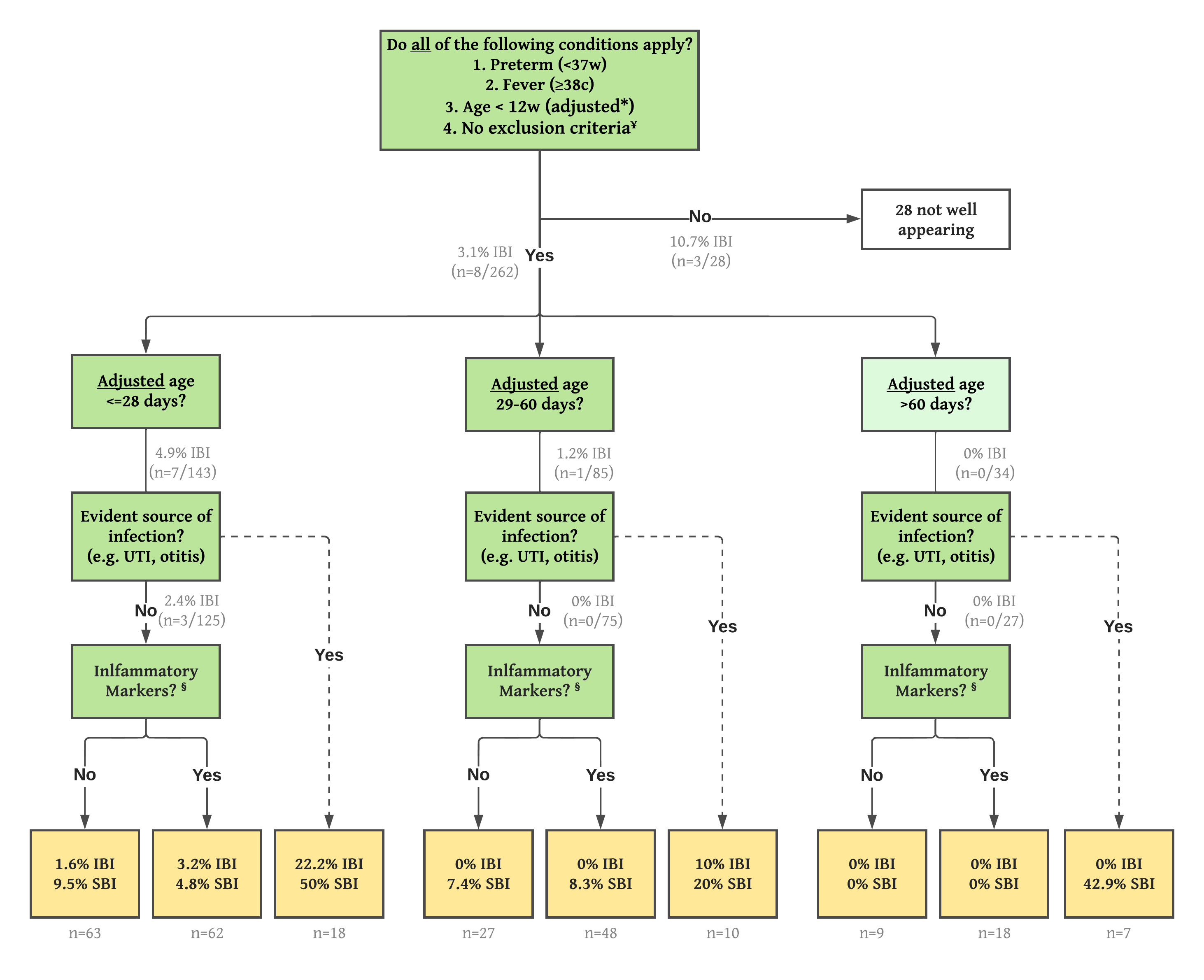


**B.**


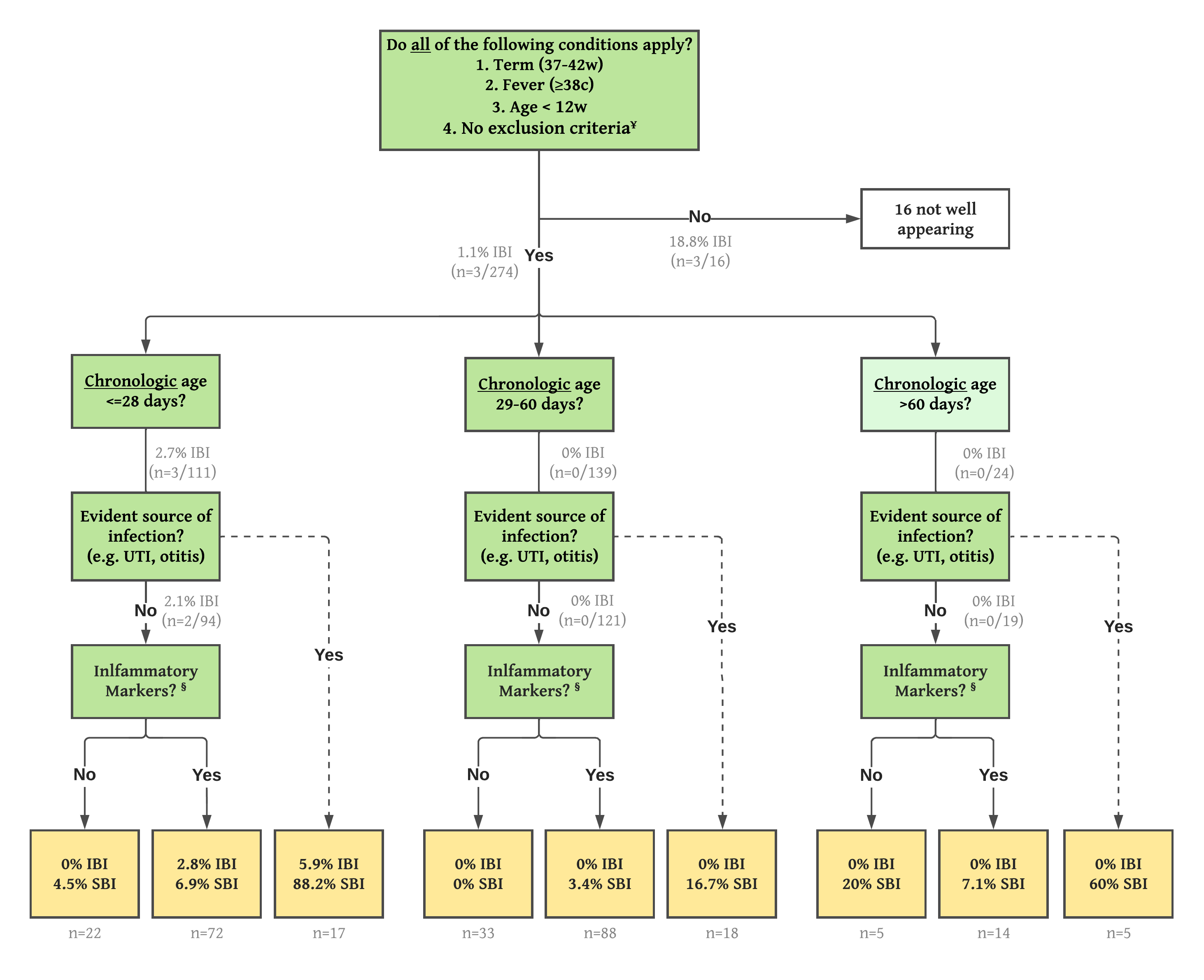


**C.**

**Appendix 4 – Interpretation of Laboratory Results**

The significance of some of the ancillary laboratory test findings in this study merit particular attention, specifically the urine dipstick, the peripheral blood neutrophil count, and the C-Reactive Protein (CRP) tests. We found the dipstick to be unreliable for detecting UTI in our cohort of ex-premature infants, with a sensitivity of only 66%. Previous studies reported dipstick sensitivity rates for detecting UTI in young infants which vary between 64% and 90%.[1,2] However, this study is the first to evaluate dipstick sensitivity in ex-premature infants. Our findings also suggest that evaluation of the CRP value and peripheral blood neutrophil percentage may improve the accuracy of risk stratification in these patients. Previous studies among full-term infants have arrived at a similar conclusion. [3,4] Nevertheless, the sample size of our cohort was not sufficient to allow determination of the role of these markers in evaluating fever in this population.

**References**

1. Reardon JM, Carstairs KL, Rudinsky SL, *et al.* Urinalysis is not reliable to detect a urinary tract infection in febrile infants presenting to the ED. *Am J Emerg Med* 2009;**27**:930–2. doi:10.1016/j.ajem.2008.07.015
2. Glissmeyer EW, Korgenski EK, Wilkes J, *et al.* Dipstick Screening for Urinary Tract Infection in Febrile Infants. *Pediatrics* 2014;**133**:e1121–7. doi:10.1542/peds.2013-3291
3. Kuppermann N, Dayan PS, Levine DA, *et al.* A Clinical Prediction Rule to Identify Febrile Infants 60 Days and Younger at Low Risk for Serious Bacterial Infections. *JAMA Pediatr* 2019;**173**:342–51. doi:10.1001/jamapediatrics.2018.5501
4. Mintegi S, Bressan S, Gomez B, *et al.* Accuracy of a sequential approach to identify young febrile infants at low risk for invasive bacterial infection. *Emerg Med J* 2014;**31**:e19-24. doi:10.1136/emermed-2013-202449

1. The definition for High Risk of SBI is derived from the Rochester Criteria and includes: History – chronic disease such as congenital heart disease, lung disease, or known genitourinary abnormality, prior hospitalizations, late discharge after delivery, **prematurity**, prior antibiotic therapy; Clinical – toxic appearance, bloody or mucoid diarrhea, focal source of infection (except for otitis media); Labs – leukocyte count bellow 5 x 10^3^/µL or over 15 x 10^3^/µL, urine with leukocytes or nitrites. [↑](#footnote-ref-1)
